# Intraductal Papillary Mucinous Neoplasm of the Pancreas: A Challenging Diagnosis

**DOI:** 10.3390/diagnostics13122015

**Published:** 2023-06-09

**Authors:** Charikleia Triantopoulou, Sofia Gourtsoyianni, Dimitriοs Karakaxas, Spiros Delis

**Affiliations:** 1 Department of Radiology, Konstantopouleio General Hospital, 14233 Athens, Greece; 2 1st Department of Radiology, School of Medicine, National and Kapodistrian University of Athens, Areteion Hospital, 11528 Athens, Greece; sgty76@gmail.com; 3 Department of Surgery, Konstantopouleio General Hospital, 14233 Athens, Greece; dimitrios.karakaksas@gmail.com (D.K.); sdelis55@hotmail.com (S.D.)

**Keywords:** intraductal papillary mucinous tumor, pancreas, CT, MRI, PETA, EUS

## Abstract

Intraductal papillary mucinous neoplasm of the pancreas (IPMN) was classified as a distinct entity from mucinous cystic neoplasm by the WHO in 1995. It represents a mucin-producing tumor that originates from the ductal epithelium and can evolve from slight dysplasia to invasive carcinoma. In addition, different aspects of tumor progression may be seen in the same lesion. Three types are recognized, the branch duct variant, the main duct variant, which shows a much higher prevalence for malignancy, and the mixed-type variant, which combines branch and main duct characteristics. Advances in cross-sectional imaging have led to an increased rate of IPMN detection. The main imaging characteristic of IPMN is the dilatation of the pancreatic duct without the presence of an obstructing lesion. The diagnosis of a branch duct IPMN is based on the proof of its communication with the main pancreatic duct on MRI-MRCP examination. Early identification by imaging of the so-called worrisome features or predictors for malignancy is an important and challenging task. In this review, we will present recent imaging advances in the diagnosis and characterization of different types of IPMNs, as well as imaging tools available for early recognition of worrisome features for malignancy. A critical appraisal of current IPMN management guidelines from both a radiologist’s and surgeon’s perspective will be made. Special mention is made of complications that might arise during the course of IPMNs as well as concomitant pancreatic neoplasms including pancreatic adenocarcinoma and pancreatic endocrine neoplasms. Finally, recent research on prognostic and predictive biomarkers including radiomics will be discussed.

## 1. Introduction

With the advances in multidetector computed tomography (MDCT) imaging and the continuing increase in overall cross-sectional imaging, pancreatic cystic lesions are more and more commonly encountered including intraductal papillary mucinous neoplasms (IPMNs) of the pancreas. The crude prevalence rate of pancreatic cystic lesions and IPMNs is 2.1% and was found to increase significantly with age in a large-scale, single-center cohort study of 21,745 asymptomatic healthy individuals [1]. In the same study, IPMNs were found to comprise 82% of incidental pancreatic cystic lesions demonstrated on CT examinations.

IPMNs arise from the epithelial lining of the pancreatic ducts. Different patterns of progression may be seen in the histopathology, from hyperplasia to invasive carcinoma [2]. They affect men and females equally and are identified usually in the 6th-7th decade [3]. Three main types may be identified: branch duct (BD) type, main duct type, subdivided into the segmental main duct (SMD) and diffuse main duct (DMD) subtypes, and mixed type (MT), combining both main types.

Imaging aims not only at the accurate diagnosis of an IPMN but also to provide information on the so-called worrisome features for malignancy. Taking into consideration the heterogenous group of lesions that this entity represents, it is of utmost importance to have in place clear diagnostic protocols and follow dedicated guidelines. The literature is extensive and, in some points, controversial concerning surgical indications, as well as the length of imaging follow-up. 

Recent research in radiomics using computed tomography (CT) and magnetic resonance imaging (MRI) has shown promise in developing prognostic and predictive biomarkers in an effort to personalize IPMN patient management. 

In this review, our aim is to focus on the challenges that IPMNs pose to the reporting radiologists, who should recognize them, as they have a pivotal role in patient management, including early implementation of appropriate therapy, namely surgical removal, allowing for cure.

## 2. Imaging Findings

**Figure 1:**
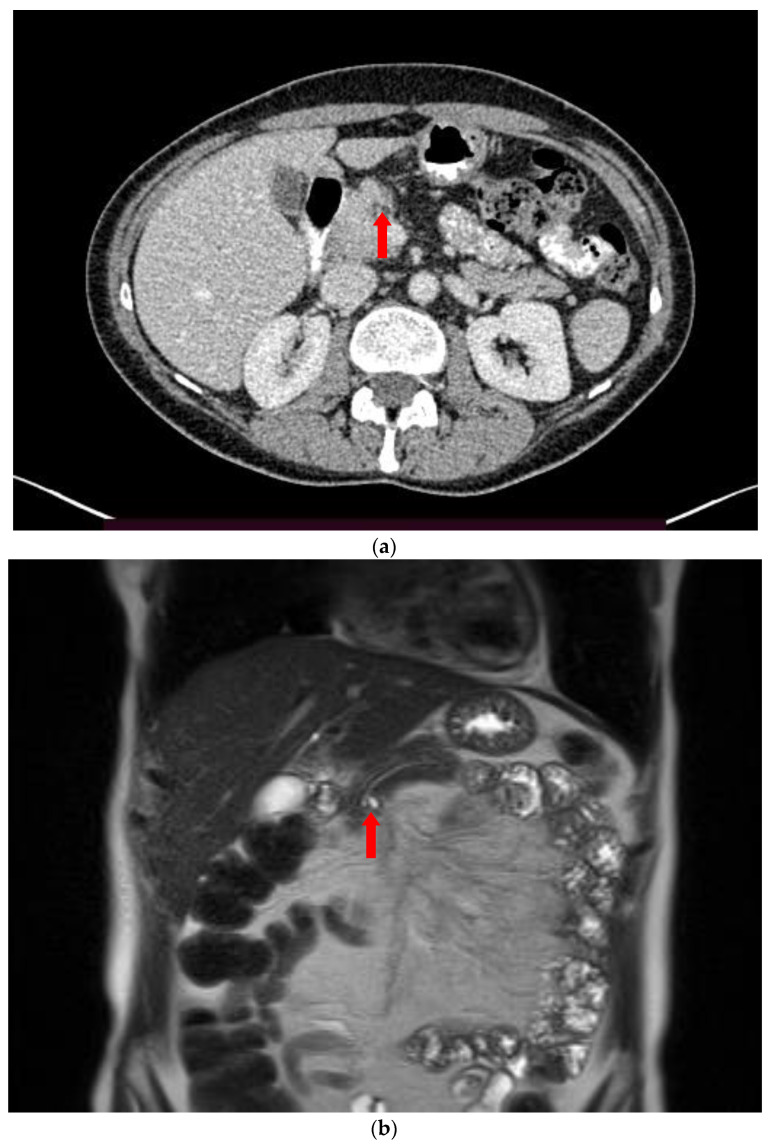
Contrast-enhanced CT image (**a**) and coronal T2-w MR image (**b**), show a small branch duct IPMN (arrow) communicating with the main duct. The IPMN is more easily seen on the MR image.

**Figure 2:**
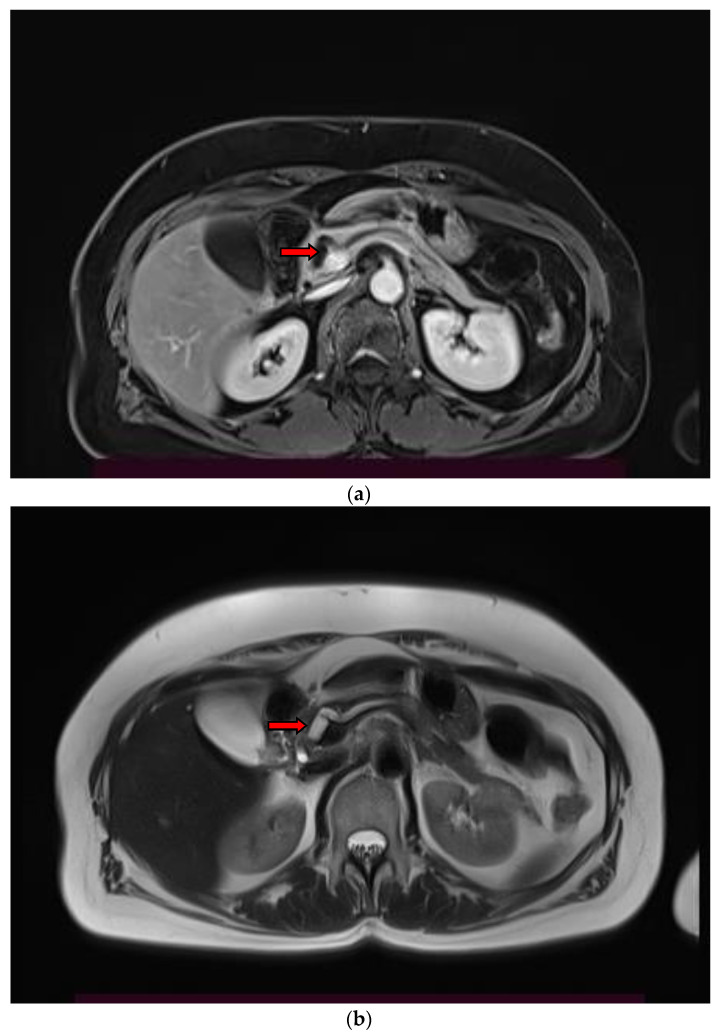
Contrast-enhanced T1-w MR image (**a**) and T2-w MR image (**b**), demonstrate a marked dilatation of the main pancreatic duct in the head and neck of the pancreas, consistent with a segmental main duct IPMN (arrow).

**Figure 3:**
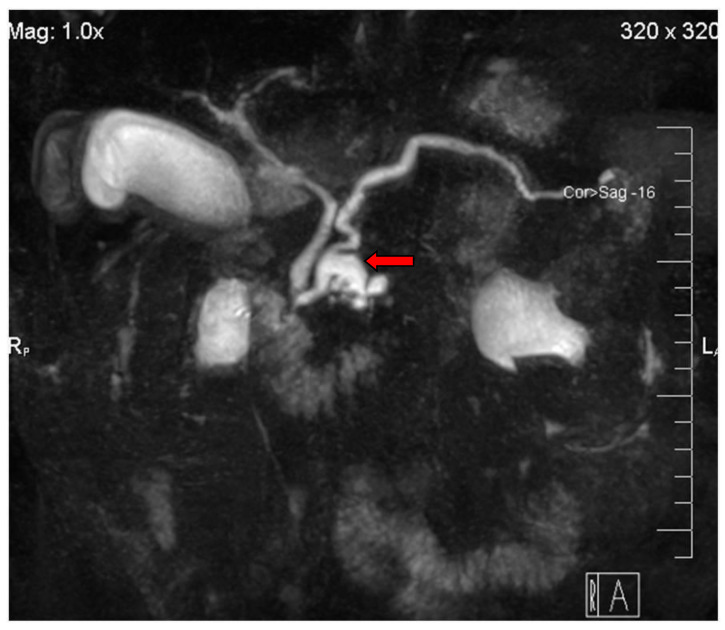
A mixed-type IPMN (arrow) is seen in the pancreatic head on this MRCP image.

A branch duct IPMN is easily recognized as a cystic dilatation of the pancreatic duct side branches. Grape-like clusters of cysts may be also seen communicating with the main pancreatic duct that is not dilated (Figure 1). A BD-type IPMN may be identified in any part of the pancreas. In diffuse main duct IPMNs, a uniform dilatation is noticed throughout the main pancreatic duct, while a patulous ampulla of Vater may be also identified. Segmental main duct IPMNs appear as a focal dilatation of the main pancreatic duct (Figure 2). Mixed IPMNs involve both the main pancreatic duct and side branches (Figure 3).

**Figure 4:**
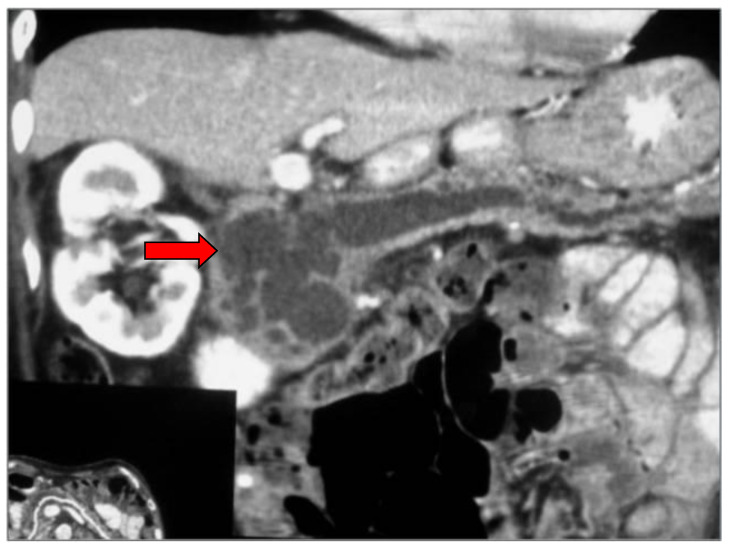
Curved reconstructed CT image in the course of the dilated pancreatic duct shows a typical case of main duct IPMN (arrow).

**Figure 5:**
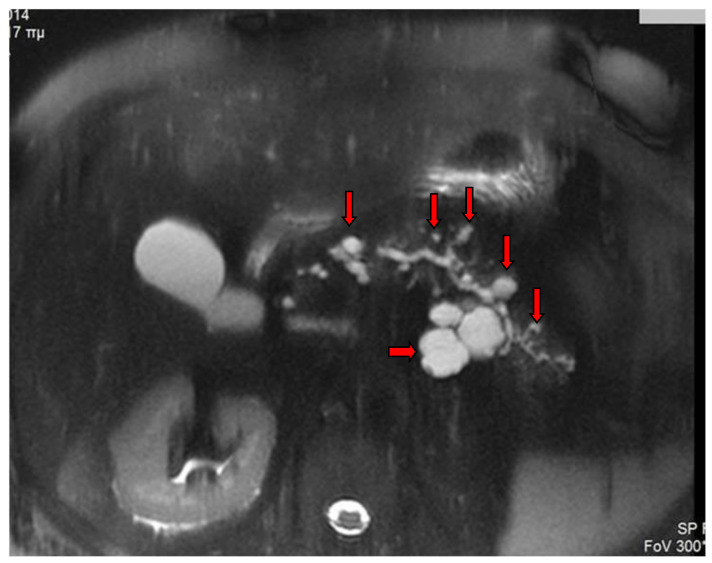
T2-w fs MR image shows many IPMNs of different sizes and morphology throughout the pancreas (arrows).

**Figure 6:**
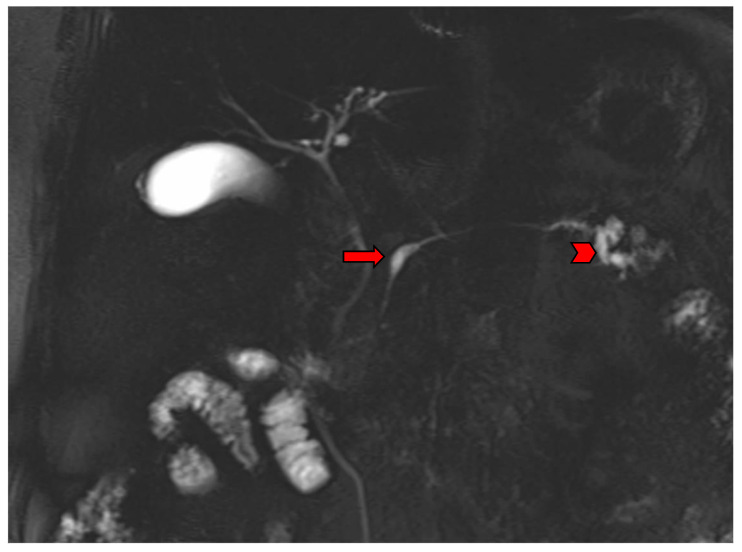
MRCP image shows a segmental main duct IPMN in the body (arrow) and a branch duct IPMN in the tail of the pancreas (arrowhead).

The main imaging characteristic of a main duct IPMN is the dilatation of the main pancreatic duct, without the presence of an obstructing lesion (Figure 4). If the diameter of the main pancreatic duct exceeds 10 mm, it is considered a high-risk stigma [4]. Cross-sectional imaging, whether MDCT or MRI, reveals the exact location of the cystic lesion, stratifies the type of IPMN, and allows for accurate measurement of the extent of the lesion. This is particularly important as the IPMN could be multifocal (Figure 5), while different types may co-exist in the same patient (Figure 6). 

**Figure 7:**
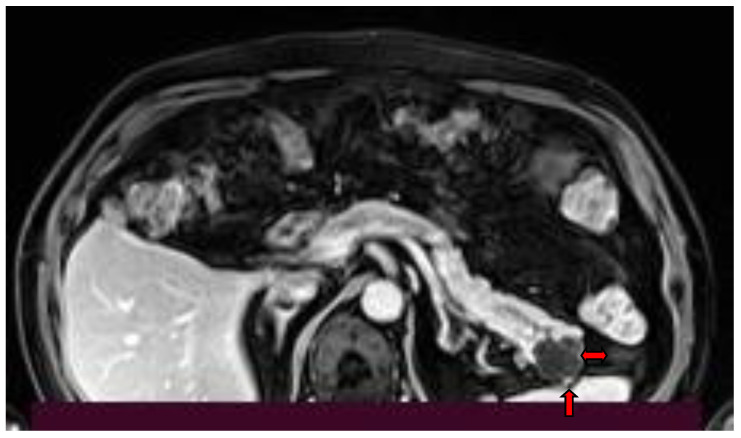
IPMN in the pancreatic tail, presenting as a cystic lesion on contrast-enhanced T1-w MR image. Enhancing mural nodules are obvious (arrows).

The duct/cyst content is hypodense in both unenhanced and after-iv-contrast-administration CT images, with fluid attenuation; while it is of high signal intensity in T2-weighted images and low signal intensity in T1-weighted images. Intraductal calcifications are rare, but when present are associated with malignant transformation [5]. Papillary growth is best appreciated after intravenous contrast administration [2] (Figure 7). Thick walls and the presence of mural nodules contribute to the irregular ductal margins in such cases. In addition to intravenous contrast enhancement, other available contrast mechanisms when performing MRI/MRCP examination of the pancreas, such as Diffusion Weighted Imaging (DWI), allow the identification of worrisome features such as enhancing mural nodule > 5 mm or thickened/enhancing cyst wall as per the international consensus guidelines for the management of IPMN published in 2017 [4]. However, the diagnosis/characterization of a mural nodule might not always be easy and endoscopic ultrasound (EUS) is recommended to simultaneously evaluate the architecture of the cyst and obtain a biopsy.

Several studies challenge the cutoff value of main pancreatic duct (MPD)  ≥  10 mm as a high-risk stigma, while a recent study investigated whether there is a different threshold of the MPD corresponding to malignancy of IPMNs according to the tumor location (head–neck versus body–tail) [6]. Different cutoff values of MPD based on CT and MRI measurements were associated with malignancy. For IPMNs involving the MPD, the threshold was found to be 8.2 mm for lesions located in the pancreatic head and neck, while for lesions in the body and tail of the pancreas, the proposed threshold was 7.7 mm.

Regarding BD type, the diagnosis and differentiation from other pancreatic cystic lesions are based on the proof of communication of the cystic lesion with the main pancreatic duct, best identified via MRI-MRCP examination. The branch duct type may be solitary or in the form of multiple cystic lesions. Solitary branch duct IPMNs are located mainly in the uncinate process and have a grape-like microcystic or a unilocular or multilocular macrocystic appearance [2] on MRCP images. Compared with 2D MRCP examination, 3D MRCP has been shown to provide better image quality, providing an improved evaluation of the pancreatic duct and morphological aspects of IPMN, being more helpful in identifying the communicating duct, in addition to being preferred for surgical planning [7].

The exploitation of the quantitative parameter of diffusion-weighted imaging (DWI) might prove to be helpful in differential diagnosis between mucinous and serous cystic tumors and in IPMN characterization (benign vs malignant). For this purpose, a spin-echo echo-planar DWI sequence with multiple b values (0, 150, 500, 1000, and 1500 s/mm^2^) was studied at 3T MRI, and different ADC values were proposed [8].

**Figure 8:**
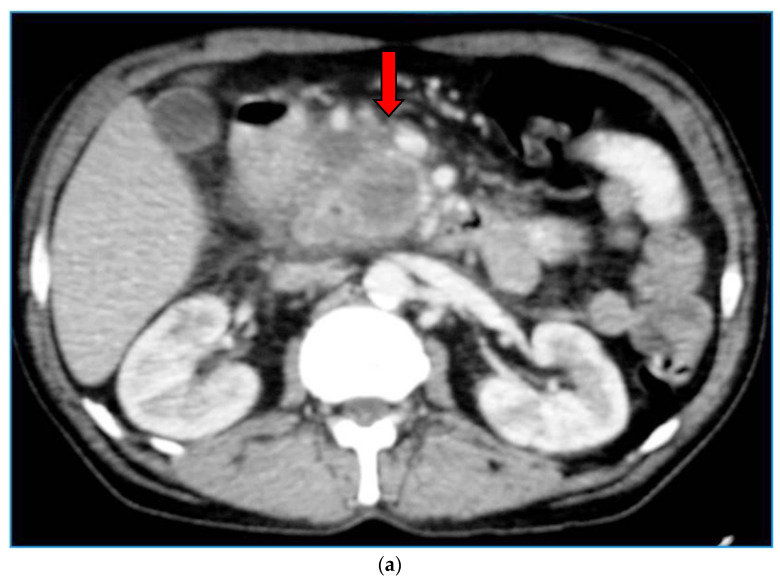

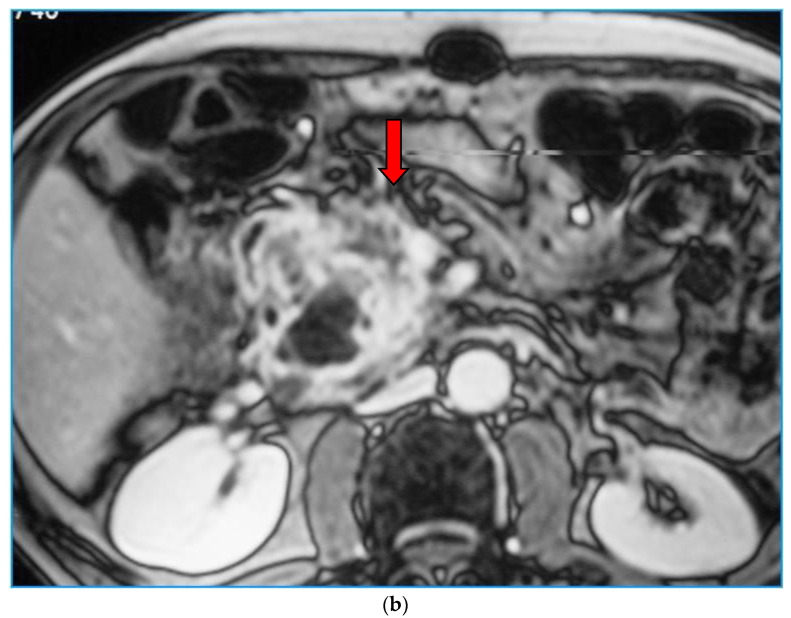
Contrast-enhanced CT image (**a**) and T1-w MR image post iv contrast administration (**b**), show an IPMN with invasive carcinoma (arrow) in the pancreatic head infiltrating the superior mesenteric vein.

A different quantitative MRI measurement that might prove to be helpful is proton density fat fraction (PDFF [%]) calculated in a multi-echo 3D DIXON sequence obtained at 3T MRI, which has been found to be significantly higher in pancreatic parenchyma of patients with intraductal papillary mucinous neoplasm with a concomitant invasive carcinoma (IPMN-IC) (Figure 8) compared to a normal pancreas group and an IPMN group without invasive carcinoma, thus serving as a potential biomarker for the malignant development of IPMN [9].

Conversely, Yamada et al. [10] demonstrated that parenchymal fatty infiltration, expressed by a lower CT density of the pancreas in patients with IPMN, may serve as an early imaging biomarker for developing malignancy in the pancreas, for both high-grade dysplasia/derived pancreatic ductal adenocarcinoma (PDAC) and for concomitant PDAC. For this purpose, the authors utilized the pancreatic index (PI) measured on CT images calculated by dividing the CT density of the pancreas by that of the spleen.

In the retrospective study of Lee et al. [11] comparing the diagnostic performance and intermodality agreement between contrast-enhanced CT and MRI for prediction of malignancy, enhancing mural nodule of ≥5 mm, abrupt main pancreatic duct caliber change, lymphadenopathy, larger main pancreatic duct size, and faster cyst growth rate were found to be more common in malignant than benign IPMNs. The diagnostic performance of contrast-enhanced CT and MRI were comparable, with good intermodality agreement. In a later study by Min et al. [12], it was shown that MRI is superior to CT for identifying IPMN-associated mural nodules. Nevertheless, diagnostic performance for differentiating malignant from benign IPMNs was found to be similar between CT and MRI.

Many studies have evaluated the usefulness of 18-fluorodeoxyglucose positron emission tomography/computed tomography (18-FDG PET/CT) in the characterization of IPMN of the pancreas. In 2019, Yamashita et al. [13] showed that 18-FDG PET accumulation was significantly related to malignancy in IPMN with a sensitivity of 0.82 and specificity of 0.71. In another systematic review [14], it was shown that 18-FDG PET/CT imaging had a very high positive and negative predictive value, as well as a specificity and accuracy of 95% and 91%, respectively in identifying malignancy (either high-grade dysplasia and/or invasive) in IPMNs.

Finally, a recent meta-analysis [15] evaluating the role of all imaging modalities in the differentiation of benign and malignant IPMNs concluded that PET/CT showed the highest AUC. MRI/MRCP and PET/CT can be used interchangeably as a first-line examination based on the overall diagnostic accuracy in the diagnosis of malignant IPMN. A negative PET/CT examination in a patient with a suspicion of a malignant IPMN on CT and/or MRI permits a safe follow-up plan and may help to avoid unnecessary surgery.

In conclusion, CT is mandatory for staging and resectability evaluation. MRI offers the advantage of a better depiction of cystic lesions and their communication with the pancreatic duct by means of MRCP imaging. Furthermore, MRI is advantageous when equivocal liver lesions need to be characterized. At the same time, PET/CT has an additional role in indeterminate IPMNs, concerning the possibility of malignant transformation, and is used in selected cases.

## 3. IPMN-Related Complications

IPMNs are frequently asymptomatic, but in some cases, epigastric pain is reported. Fistulas may develop between IPMNs and various organs [16,17]. Their presence is usually related to invasive carcinoma and may involve the duodenum (64%), the common bile duct (56%), or the stomach (17%). The hypothesized mechanism for the formation of pancreaticogastric and pancreaticoduodenal fistulas is by contiguous invasion [18]. In cases of benign IPMNs, the mechanism differs and is associated with the increase of the intraductal pressure due to the presence of dense mucin causing ductal dilatation and wall disruption. Chronic inflammatory infiltration of the pancreatic tissue has been described as another possible mechanism in these cases [19].

Another rare complication that has been reported is pseudomyxoma peritonei caused by a ruptured pancreatic duct and mucin spread in the peritoneum [20]. The duct rupture and the subsequent mucin spread is a serious event as it is related to tumor seeding. Intraductal hemorrhage has also been reported in some cases, detected as high attenuation content on unenhanced CT images or as high signal intensity on fat-suppressed T1-weighted MR images. Intraductal hemorrhage may also lead to perforation and fistula formation [21].

Acute pancreatitis (AP) may also complicate an IPMN with a rate that varies from 12 to 67% [22]. The rate of AP does not seem to differ among benign and malignant IPMNs. Patients with IPMN are more prone to develop acute pancreatitis than patients with pancreatic adenocarcinoma because of the obstruction of the main duct by thick, abundant mucin and marked ductal dilatation. It is important to recognize an IPMN in cases of repeated episodes of AP, as these patients require surgery. The differential diagnosis between IPMN and AP-related pseudocysts is often challenging.

In patients with no high-risk stigmata who develop recurrent acute pancreatitis, endoscopic sphincterotomy could be offered as a minimally invasive technique to reduce the rate of these episodes. In selected patients, it can be a safe and effective treatment, without obviating the need for follow-up.

IPMNs are considered one of the main precursor lesions of pancreatic ductal adenocarcinoma (PDAC) following the tumoral intraepithelial neoplasm pathway. When an IPMN progresses to an invasive PDAC, it is referred to as an “IPMN with an invasive carcinoma” or “intraductal papillary mucinous carcinoma” (IPMC). IPMCs have distinct molecular, biological, and prognostic characteristics and account for about 10% of resected pancreatic cancers of ductal origin [23]. It is of utmost importance to differentiate IPMN-associated adenocarcinomas from other pseudo-IPMN lesions that can be secondary duct ectasias, retention cysts, large-duct-type PDACs, pseudocysts, cystic tumor necrosis, simple mucinous cysts, groove pancreatitis-associated paraduodenal wall cysts, or congenital cysts [24].

## 4. IPMN Management Guidelines

Currently, three primary guidelines exist regarding the management of the continuously increasing number of diagnosed IPMN cases. All three try to answer the critical question of “to operate or to follow up”, as this poses a critical dilemma faced by involved clinicians. The guidelines are based on objective imaging, laboratory, and clinical findings to reduce the number of unnecessary pancreatic operations and their associated morbidity and mortality rates.

These are the International Association of Pancreatology Guidelines first published in 2006 and revised in 2012 and 2017, the European evidence-based Guidelines published in 2013 and updated in 2018, and the American Gastroenterology Association Guidelines published in 2015. All agree on the main surgical intervention indications categorized as “Worrisome Features-Relative Indications” or “High Risk Stigmata-Absolute Indications” of a branch duct IPMN [4,25,26] (Table 1).

Worrisome features or relative indications according to the International Association of Pancreatology and European guidelines, respectively, cannot be solely used to predict the malignant course of an IPMN neoplasia. Patients with worrisome features had a significantly better 5-year disease-specific survival compared with those with high-risk stigmata (96% vs. 60%, respectively). The surgical intervention indications also differ between the guidelines, as the European guidelines warrant surgical resection in cases where there is one relative indication in surgically fit patients. In contrast, the International Association of Pancreatology guidelines require the presence of at least one worrisome feature leading to further diagnostic evaluation with EUS to better assess the risk of malignancy. The American Gastroenterology Association guidelines recommend surgery for cases presenting with an MPD ≥ 5 mm and the presence of a solid component or cytology positive for malignancy [4,25,26].

“Worrisome features” on imaging include a cyst of 3 cm, an enhancing mural nodule < 5 mm, thickened enhancing cyst walls, main pancreatic duct dilatation with a diameter 5–9 mm, abrupt change in the caliber of the main pancreatic duct with distal pancreatic atrophy, lymphadenopathy, an elevated serum level of carbohydrate antigen (CA)19-9, and a rapid rate of cyst growth > 5 mm/2 years. The evaluation of these patients by endoscopic ultrasonography (EUS) using techniques such as Doppler EUS or contrast-enhanced harmonic EUS is mandatory. The presence of blood supply in a mural nodule can further stratify the lesion and it is important for patient management [4].

Conversely, the presence of “High-risk Stigmata” on CT, MRI, or EUS (i.e., obstructive jaundice in a patient with a cystic lesion of the pancreatic head, enhancing mural nodule of 5 mm, or main pancreatic duct diameter of 10 mm) is a clear indication for lesion resection in surgically fit patients without further testing. This is based on the high frequency of invasive carcinoma and high-grade dysplasia in MD-IPMN, which is 61.6% (range, 36–100%), while the mean frequency of invasive IPMN is 43.1% (range, 11–81%). Precursor lesions of pancreatic cancer such as IPMNs with HGD, pancreatic intraepithelial neoplasia 3 (PanIN 3), and mucinous cystic neoplasia (MCN) with HGD should be resected as this is the only chance for patient cure [4].

Given the higher malignant potential of main duct and mixed-type IPMNs, an early surgical excision decision in surgically fit patients can be justified [27,28]. All patients with cysts of 3 cm in size without “Worrisome Features” should undergo surveillance according to size stratification [4]. Additionally, the Shin score published in 2010 is a complimentary tool that comprises five variables, namely, age, history of pancreatitis, CA19-9 level, pancreatic duct diameter, and mural nodules. Patients with a Shin score of <1 warrant surveillance, while those with a score of >4 should undergo surgery. There are no clear guidelines for the treatment of patients with Shin scores of 2 or 3, which should be individualized [29].

Given the growing number of incidentally diagnosed cases in asymptomatic patients, a dilemma will frequently arise regarding therapeutic handling. Symptomatic patients may have a higher likelihood of malignancy or complications. Surgical intervention is often recommended, especially if the IPMN has high-risk features such as large size, main pancreatic duct involvement, or worrisome imaging findings. However, symptomatic patients may “deserve” more conservative management, especially in cases when there is a lack of high-risk features. Surveillance and regular imaging follow-up are usually recommended. This allows for the detection of any changes in the IPMN that may warrant intervention. In the presence of high-risk features, though, surgical intervention may still be considered. Eventually, the decision of how to proceed has to be, as much as possible, individualized and be taken in the context of a multidisciplinary team discussion.

In conclusion, following the initial evaluation with detailed patient history, physical examination, and laboratory tests, imaging studies should assess the size, location, and morphology of the IPMN and identify any high-risk or worrisome features. A multidisciplinary team will then evaluate the patient’s symptoms and overall health and discuss the patient’s clinical situation, risk factors, and potential management options. According to the aforementioned guidelines, asymptomatic, low-risk IPMN cases should be followed-up every 6–12 months, asymptomatic, high-risk IPMN cases should be considered for surgical intervention after evaluating the patient’s overall health and risk-benefit analysis, and symptomatic IPMN cases should be offered a surgical intervention, especially if high-risk features are present. When resection takes place, the pathology report will direct the further type and timeframe of the follow-up plan.

## 5. EUS-FNA Added Value for IPMN Stratification

The added value of EUS-FNA and cytological interpretation is well recognized, especially for the evaluation of a small BD-IPMN without worrisome features. The recognition of a high-grade epithelial atypia, although insufficient for a malignant interpretation, represents a more sensitive predictor of invasive carcinoma or HGD than positive cytology [4,26]. The presence of cellular atypia in epithelial cells in the cyst fluid shows an 80% accuracy in the prediction of invasive carcinoma and HGD in a mucinous cyst, while more cancers can be detected in small IPMNs than the use of “worrisome features”. Concerning molecular analysis of the cyst fluid, although still evolving, it is crucial for the recognition of a mucinous cyst. There is evidence that the detection of KRAS mutations supports accurately the diagnosis of a mucinous, but not necessarily malignant, cyst. Recent studies have evaluated the role of GNAS mutations, showing that this may help in distinguishing mucinous cysts from benign cysts [30,31]. Patients with positive fluid obtained cytology should be considered for surgery [32].

## 6. Surgical Considerations Regarding IPMN Management

Referral to a high-volume center for IPMN cases is without any doubt the cornerstone of their management, given the high level of complexity and experience required for their correct diagnosis and follow-up, or even more important, the technical difficulty and postoperative management of the required surgical operations and different types of resections [33,34,35].

IPMNs are considered in most cases as pre-malignant neoplasms and their resection must follow standard oncological principles such as a negative pathology report of the resection margin and proper lymphadenectomy [36]. Moreover, IPMNs can be characterized as the result of a pancreatic “field defect” because there is a possibility that all pancreatic ductal epithelial cells are at risk of dysplastic change [37].

Carbohydrate antigen (CA) 19-9 is a tumor biomarker related to pancreatic adenocarcinoma. When elevated in cases of IPMN, it is considered a worrisome feature in current guidelines and a relative criterion for resection. This biomarker has a prognostic value as CA 19-9 levels > 37 U/mL are associated not only with an increased likelihood of invasive carcinoma but also with worse overall and disease-free survival.

There are only three types of pancreatic resection that can assure the best oncological postoperative outcomes. These are pancreatoduodenectomy, distal pancreatectomy, and total pancreatectomy. Limited resections are considered only in a case-by-case setting and for a small minority of cases and they can be performed as focal non-anatomic excision or enucleation [38]. Cases of BD-IPMN without clinical, radiologic, cytopathologic, or serologic suspicion of invasive carcinoma may be treated in such a way. They are associated sometimes with leakage of mucin followed by pseudomyxoma peritonei and present with a higher rate of pancreatic fistulae and higher recurrence percentages from potential residual neoplasm [39,40,41].

Pancreatoduodenectomy is indicated in cases of dilatation of the main pancreatic duct in the head of the pancreas or the uncinate process, while lesions in the body and tail of the organ are treated with distal pancreatectomy with or without splenic preservation. Total pancreatectomy is the treatment of choice for diffuse multifocal disease, familial history of pancreatic adenocarcinoma, and failure to achieve a negative resection margin due to high-grade dysplasia in repeated intraoperative frozen sections. Total pancreatectomy postoperative sequelae are considered much more acceptable than the high risk of carcinoma development. However, due to long-term postoperative outcomes, total pancreatectomy cannot be advocated for all IPMNs even if multiple, and should only be offered in younger patients who can better handle the consequences of diabetes and exocrine insufficiency [42,43].

The intraoperative frozen section of the resection margin is a prerequisite for a safe oncologic outcome in cases when a partial pancreatectomy is attempted. The extent of the resection is based solely on its findings. High-grade dysplasia and carcinoma warrant extension of the resection or even total excision of the organ, but low-grade dysplasia is a finding that does not need any further modification of the procedure [44].

Moreover, it is interesting and at the same time important from an oncological outcome perspective, that T1a invasive intraductal papillary mucinous carcinoma has an excellent postoperative survival course, in cases with no lymphatic involvement, compared to pancreatic ductal adenocarcinoma, probably because of the more indolent behavior of this type of neoplasia [45].

## 7. Histopathological Correlation

IPMNs are characterized by abundant mucinous production and proliferation of mucinous epithelial cells resulting in papillary epithelial growth. Intraductal oncocytic papillary neoplasms (IOPNs), and intraductal tubulopapillary neoplasms (ITPNs) were considered variants of IPMN. In the 5th edition of the World Health Organization (WHO) Report, published in 2019, IOPNs and ITPNs are classified as distinctive neoplasms.

A combination of differentiations can be displayed in IPMNs using immunolabeling. The main differentiations are related to the neoplastic epithelium, which can be gastric-foveolar, intestinal, and pancreatobiliary. Gastric–foveolar differentiation is the most common type and is mainly seen in BD-IPMNs. Intestinal differentiation is the second most common type and is characterized by the presence of long finger-like (villous) papillae. Intestinal-type IPMN is the precursor of all colloid carcinomas of the pancreas.

The identification of the various types of differentiation in IPMNs is important but not as clinically significant as the degree of dysplasia assigned to them [46]. The grading system is simplified in low- vs. high-grade. Grading of dysplasia has clinical importance as high-grade neoplasms are more likely to have an associated invasive carcinoma. The presence of high-grade dysplasia in resected neoplasms has a major prognostic and predictive value as it is associated with a higher risk of progression in the remnant pancreas after surgery [47,48].

## 8. Concomitant Pancreatic Neoplasms

Patients with IPMN are at a well-recognized risk of developing concomitant pancreatic adenocarcinoma (PDAC) [49]. The term concomitant PDAC with IPMN refers to a PDAC that is separated from the IPMN by an uninvolved segment of the pancreatic duct and with no clearly visible areas of transition in between.

There are many retrospective studies reporting cases of patients with IPMN developing adenocarcinoma in areas not related to pancreatic cysts, with incidences of between 4% and 11% [50,51,52,53,54,55,56,57]. Adenocarcinoma may develop many years after the diagnosis of IPMN. In a prospective study, which followed 89 patients with IPMN over a 17-year period, four developed concomitant pancreatic adenocarcinoma [58]. This fact supports the role of long-term follow-up in patients with IPMNs.

It is quite difficult to differentiate pancreatic adenocarcinoma from malignant IPMN as they share almost the same imaging characteristics. The only criterion that may be used for the differential diagnosis is that in cases of pancreatic adenocarcinoma with obstruction of the pancreatic duct, there is upstream ductal dilatation and atrophy of the parenchyma. Conversely, in cases of IPMN, the duct is dilated but there is no parenchymal atrophy. As both cases need surgery, accurate differential diagnosis is not mandatory, and the radiologist should focus on resectability evaluation using the same criteria as for pancreatic cancer.

Identical genetic alterations can well explain the co-existence of IPMN with PDAC. Somatic-activating mutations in the KRAS and GNAS genes have been described in both diseases and are the most common alterations. There is increasing evidence that PDACs in the vicinity of IPMN lesions are often significantly smaller and have a different biological behavior compared to PDACs with no associated IPMN, being less aggressive and associated with longer survival [59].

Another important observation supported by the literature is that the occurrence of IPMNs in association with pancreatic neuroendocrine tumors (PNETs) is more frequent than expected [60]. Patients with concomitant IPMN and PNET have been reported in many studies at a rate of 2.8% and 4.6% of all cases [61,62,63]. Goh et al. [61] hypothesized that this association could be related to the existence of common underlying risk factors and genetic alterations.

There are two proposed hypotheses for the tumorigenesis of concomitant PNET and IPMN published: (1) one cell type in a unique tumor could transdifferentiate into another cell type; and (2) two cell types could arise from a common neoplastic progenitor; the latter hypothesis being supported by many investigators.

Terada et al. [64] suggested that IPMN has the potential for endocrine differentiation. They found that argentaffin-serotonin- and gastrin-secreting cells were present in IPMN but not in normal pancreatic ductal cells. However, Hashimoto et al. [65] studied a case of mixed PNET and IPMN and found positivity for exocrine markers expressed on some endocrine tumor cells, supporting the contention that the endocrine tumor cells might transdifferentiate to ductal tumor cells.

Some reports suggest that there is a lack of awareness of the potential for the concomitance of PNET and IPMN, thus leading to the poor examination of specimens and a high rate of underreported or undetected cases [66]. Therefore, it is important that radiologists and pathologists are aware of the possibility of concomitant IPMN with PNET in order to recognize these neoplasms early, as this may certainly affect patient management and prognosis.

## 9. Advanced Endoscopic Procedures

EUS-guided needle confocal laser endomicroscopy (nCLE) is a novel technique that permits real-time microscopic imaging of intra-cystic epithelium allowing in vivo pathological analysis of pancreatic cystic lesions (PCLs). Characteristic features of IPMNs have been well established in recent literature.

In a recent meta-analysis (7 studies and 324 patients), this technique was evaluated for the differential diagnosis between mucinous and non-mucinous cystic pancreatic lesions. The sensitivity, specificity, and accuracy were 85% (95%CI: 71–93%), 99% (95%CI: 90–100%), and 99% (95%CI: 98–100%), respectively, while the risk of post-procedure acute pancreatitis was only 1% [67].

The INDEX study was designed as a post hoc analysis to identify multiple nCLE imaging variables that would detect advanced neoplasia in IPMNs [68]. The variables with the highest inter-observer agreement according to this study were papillary epithelial thickness and darkness. Specifically, papillary epithelial thickness visualized by nCLE (width ≥ 50 μm) had a specificity of 100% (95%CI: 69–100%) for the detection of advanced neoplasia. Additionally, estimation of the papillary epithelial darkness (cut-off ≤ 90 pixel intensity) revealed a specificity of 100% (95%CI: 69–100%), while the sensitivity and AUC were reported to be 87.5% (95%CI: 62–99%) and 0.90, respectively [68].

However, we should mention the potential limitations of nCLE. Manually evaluation of papillary epithelial thickness and darkness may lead to differences in the inter-observer interpretation of images. The development of an artificial intelligence model has partially solved these issues and identified advanced neoplasia in IPMNs with a sensitivity of 83% and specificity of 88%, which are above the minimum levels of the Fukuoka or AGA guidelines [69].

There is clear evidence that nCLE is an important diagnostic technique. Despite this, its incorporation into the diagnostic clinical routine is still lacking. High equipment costs are the main limitation, while continuous training in image acquisition and interpretation is definitely needed. Complications such as acute pancreatitis are still slightly higher than the standard EUS-FNA examination and care should be taken for their prevention.

## 10. Molecular Biomarkers

The introduction of next-generation sequencing (NGS) offers a new possibility in the accurate diagnosis and classification of pancreatic cystic lesions (PCLs). Small gene panels or whole exome NGS can be used. By these methods, the evaluation of intact cell and cell-free nucleic acid is allowed, as these can be found in the cyst fluid. The presence of DNA mutations (KRAS, CDKN2A, SMAD4, PTEN, PIK3CA, and TP53) can be associated not only with pancreatic adenocarcinoma but also with mucinous cystic lesions and IPMNs.

Molecular analysis of cyst fluid is of utmost importance as it can contribute to the risk estimation of IPMNs. In a large meta-analysis based on six studies, McCarty et al. [70] reported that the presence of KRAS and GNAS mutations succeeded in the detection of mucinous PCLs with a very high specificity of 99% (95%CI: 67–100%), and also a high diagnostic accuracy of 97% (95%CI: 95–98%). Additionally, dual KRAS/GNAS mutation had 94% sensitivity, 91% specificity, and 97% accuracy for diagnosing IPMNs. Recently, Ren et al. found that uncommon BRAF mutations are characteristics of a significant subset of IPMNs that lack KRAS mutations. These observations indicate that RAS-MAPK dysregulation is often found in these tumor types [71].

Risk stratification of IPMNs is quite challenging. Singhi et al. [72] used next-generation sequencing in order to evaluate DNA mutations in IPMNs that were associated with advanced neoplasia. They analyzed 102 patients with a proven diagnosis by histopathology. The presence of TP53, PIK3CA, and/or PTEN mutations was evaluated. Ultimately, they reported 88% sensitivity and 95% specificity in the diagnosis of IPMN-related advanced neoplasia.

Cyst fluid molecular analysis by next-generation sequencing presents many advantages compared to measuring cyst CEA levels, which is the standard technique, with better accuracy, and furthermore, offers the possibility of providing risk estimation for IPMNs. However, it is not readily available and the high cost represents a financial barrier to universal adaptation. These advanced techniques should be used in centers with high expertise in the management of cystic pancreatic lesions.

## 11. Radiomics–Future Directions

Despite improvements in imaging, the morphologic features of IPMNs on CT and/or MRI are still not clear enough to assess dysplasia. This is the reason why even the most recently revised guidelines may lead to unnecessary pancreatic surgery in benign cases. Lekkerkerker et al. conducted a very important study in an effort to compare the ICGs (2012) with the European (2013) and American guidelines (2015) and evaluated their performance on 75 patients with histopathologically proven IPMNs. It was shown that surgery was justified in only half of the cases (54%, 53%, and 59% of the patients, respectively) [73].

There is obviously a need to develop new biomarkers that could non-invasively assess the risk of malignancy with greater accuracy in the heterogenous group of IPMNs. Radiomics is based on data mining from medical images in order to combine qualitative and quantitative information [74]. It is based on the computerized extraction of quantifiable data from radiological images from different sources. Through this extraction, mineable databases are created that can be used for diagnosis assessment, prognosis estimation, and prediction evaluation.

Three are not many studies that have evaluated the role of radiomics in differentiating IPMNs with high-grade dysplasia (HGD) or advanced neoplasia from indolent lesions with low-grade dysplasia (LGD) [75,76,77,78]. In most of these studies, CT scans were used and patients with confirmed surgical histopathology were included. A recent multicenter study by Cui et al. [79], presents a radiomic nomogram based on MRI to predict high-grade dysplasia or adenocarcinoma in branch duct IPMNs. Clinical variables were combined with a radiomic signature that incorporated nine features. Their results were promising as the predictive nomogram they created diagnosed advanced neoplasia with AUC values of 0.903 (in the training cohort; with a sensitivity of 95% and a specificity of 73%), and 0.884 (in one of two external validation cohorts; with a sensitivity of 79% and a specificity of 90%) [79].

In a more recent study, the authors used contrast-enhanced CT-based radiomics to differentiate between LGD, HGD, and invasive IPMN. A large retrospective series of 408 consecutive patients with histologically proven IPMN after resection was used [80]. They showed many, and significant, differences in the training cohort between patients with benign and malignant IPMNs (in 85/107 radiomic features). The results were really promising considering that the multivariate model differentiated benign from malignant tumors in the training cohort with an area under the ROC curve (AUC) of 0.84, a sensitivity of 0.82, and a specificity of 0.74, while in an external validation cohort, the scores were an AUC of 0.71, a sensitivity of 0.69, and a specificity of 0.57. Thus, preoperative CT-based radiomic analysis has the potential to differentiate benign from malignant IPMNs.

There is no doubt that radiomics represent a promising non-invasive tool for the accurate classification and risk estimation of pancreatic cystic lesions and, specifically, IPMNs and will impact proper patient management. Radiomics has so far demonstrated a great potential for diagnosis, prognosis, and risk evaluation in pancreatic neoplastic cysts, providing prognostic and predictive biomarkers. However, it is still a novel technique and has been used to date mainly for clinical trials in academic centers. Protocol standardization is challenging as it may affect all steps of image analysis [81].

## 12. Conclusions

IPMNs are classified into three subtypes MD-IPMN, BD-IPMN, and mixed-type. Each subtype has different clinical/imaging presentation and management strategies, which further adds to the diagnostic complexity. The management of IPMN is dependent on risk stratification based on various factors including size, extension, and the presence of dysplasia. Imaging ambiguity remains a challenge in both diagnosis and further stratification of this entity. Advances in radiomics are expected to provide tumor-specific treatment strategies and optimize patient management in the future.

It is of utmost importance for radiologists to be familiar with current guidelines and to participate in multidisciplinary meetings, as each case requires a tailored approach and personalized treatment might be offered. There is a great need to reduce the rate of futile surgeries without missing the therapeutic window in selected cases. Radiologists will have an increasing role in the chain of patient management and should adapt new techniques in their daily clinical practice. Precise diagnosis should also contain detailed information including proposing prognostic and predictive imaging biomarkers and providing guidance for the best patient treatment.

## Figures and Tables

**Table 1:** Surgical intervention indications.

**High-Risk Stigmata**
enhancing solid component ≥ 5 mm
main pancreatic duct ≥ 10 mm
obstructive jaundice
**Worrisome Features**
thickened and enhancing cyst wall
enhancing mural nodule < 5 mm
main pancreatic duct 5–9 mm
cyst ≥ 3 cm
lymphadenopathy
abrupt change in caliber of the pancreatic duct with distal pancreatic atrophy
cyst growth rate ≥ 5 mm in two years
elevated CA 19-9

## Data Availability

No new data were created or analyzed in this study. Data sharing is not applicable to this review article.
